# Fluoroquinolone-Resistant Enteric Bacteria in Sub-Saharan Africa: Clones, Implications and Research Needs

**DOI:** 10.3389/fmicb.2016.00558

**Published:** 2016-04-22

**Authors:** Marie A. Chattaway, Aaron O. Aboderin, Kayode Fashae, Chinyere K. Okoro, Japheth A. Opintan, Iruka N. Okeke

**Affiliations:** ^1^Gastrointestinal Bacteria Reference Unit, Public Health EnglandLondon, UK; ^2^Department of Medical Microbiology and Parasitology, College of Health Sciences, Obafemi Awolowo UniversityIle-Ife, Nigeria; ^3^Department of Microbiology, University of IbadanIbadan, Nigeria; ^4^Department of Medicine, University of CambridgeCambridge, UK; ^5^Department of Medical Microbiology, School of Biomedical and Allied Health Sciences, University of GhanaAccra, Ghana; ^6^Department of Pharmaceutical Microbiology, Faculty of Pharmacy, University of IbadanIbadan, Nigeria

**Keywords:** fluoroquinolone resistance, quinolone resistance, antimicrobial resistance, Africa, Escherichia coli, Salmonella, *Vibrio cholerae*, Campylobacter

## Abstract

Fluoroquinolones came into widespread use in African countries in the early 2000s, after patents for the first generation of these drugs expired. By that time, quinolone antibacterial agents had been used intensively worldwide and resistant lineages of many bacterial species had evolved. We sought to understand which Gram negative enteric pandemic lineages have been reported from Africa, as well as the nature and transmission of any indigenous resistant clones. A systematic review of articles indexed in the Medline and AJOL literature databases was conducted. We report on the findings of 43 eligible studies documenting local or pandemic fluoroquinolone-resistant enteric clones in sub-Sahara African countries. Most reports are of invasive non-typhoidal *Salmonella* and *Escherichia coli* lineages and there have been three reports of cholera outbreaks caused by fluoroquinolone-resistant *Vibrio cholerae* O1. Fluoroquinolone-resistant clones have also been reported from commensals and animal isolates but there are few data for non-Enterobacteriaceae and almost none for difficult-to-culture *Campylobacter* spp. Fluoroquinolone-resistant lineages identified in African countries were universally resistant to multiple other classes of antibacterial agents. Although as many as 972 non-duplicate articles refer to fluoroquinolone resistance in enteric bacteria from Africa, most do not report on subtypes and therefore information on the epidemiology of fluoroquinolone-resistant clones is available from only a handful of countries in the subcontinent. When resistance is reported, resistance mechanisms and lineage information is rarely investigated. Insufficient attention has been given to molecular and sequence-based methods necessary for identifying and tracking resistant clones in Africa and more research is needed in this area.

## Introduction

### Recent trends in fluoroquinolone use and resistance in africa

The antibacterial activity of nalidixic acid, a derived by-product from the synthesis of the antimicrobial chloroquine, was discovered in the 1960s. This first quinolone was the base molecule used to synthesize the even more active fluoroquinolones, which also had more favorable pharmacokinetics (Mitscher, 2005). First generation fluoroquinolones (second generation quinolones), like ciprofloxacin, became clinically available in the 1980s and were patent protected until the 2000s (Paton and Reeves, 1988). Until recently, ciprofloxacin was the most potent fluoroquinolone and highly active against virtually all bacterial enteropathogens. It was therefore widely considered to be the drug of choice for patients with enteric fever, severe gastroenteritis, cholera and salmonellosis (Brown and Reeves, 1997). These and other enteric infections are common in the sub-Saharan African sub-continent where an estimated 960–980 million people, most of them young, share the highest risk of infectious disease. Within a year of introduction of ciprofloxacin, resistance appeared and soon became commonplace (Brown and Reeves, 1997). However, 30 years since its introduction, ciprofloxacin is still the most commonly prescribed fluoroquinolone along with levofloxacin, ofloxacin, and moxifloxacin (Redgrave et al., 2014).

Being orally active, broad-spectrum, and heat-stable, the potential for fluoroquinolone application as well as misuse was high. When the efficacy of earlier orally active antibacterials such as the tetracyclines, amino penicillins, and trimethoprim-sulphonamide combinations declined due to rapidly rising antimicrobial resistance rates, fluoroquinolones were recognized as effective alternatives for common and life-threatening infections (Green and Tillotson, 1997). They quickly became among the most widely applied antimicrobials worldwide. The initial high cost of these drugs prohibited their use in the least affluent African settings. The situation changed upon ciprofloxacin patent expiry in 2003, when cheap generics entered African markets. Other fluoroquinolones, such as pefloxacin, met similar fates, and the fluoroquinolones rapidly became first-line drugs for the empiric treatment of many life-threatening infections (Orogade and Akuse, 2004).

The WHO recently highlighted fluoroquinolone resistance in *Escherichia coli* and related organisms as a principal public health threat (WHO, 2014). Fluoroquinolone resistance was rare worldwide in the 1980s. In Nigeria, as elsewhere, rapid escalation of fluoroquinolone use has been paralleled by a similarly rapid increase in the prevalence of resistant bacteria, including enterics (Omigie et al., 2009; Lamikanra et al., 2011; Namboodiri et al., 2011). In part because quinolone resistance evolved later but also perhaps because mechanisms conferring resistance to a wide range of drugs may have predominated before quinolone-specific mechanisms evolved, fluoroquinolone-resistant strains are typically resistant to multiple antimicrobials (Lamikanra et al., 2011; Namboodiri et al., 2011). Other factors may account for association of fluoroquinolone resistance and multiple resistance in Africa in particular. Quinolones were widely used for decades elsewhere in the world so that resistance genes and resistant clones had already evolved by the time fluoroquinolones were significantly employed in Africa. It has been hypothesized that chloroquine contributed to selective pressure for fluoroquinolone-resistant bacteria in malaria endemic settings but available data do not support this hypothesis for Nigeria (Davidson et al., 2008; Lamikanra et al., 2011). Irrespective of other factors driving fluoroquinolone resistance in enterics across Africa, introduction of fluoroquinolones into routine care in African clinics rapidly enhanced their selection and transmission in a paradigm that is instructive for future antibacterial introductions. In this paper, we review what is known about fluoroquinolone resistant enteric clones in Africa.

### Fluoroquinolone resistant enteric organisms and overview of clonal expansion problem

Among enteric bacteria, fluoroquinolone resistance has predominantly been reported in the literature on *E. coli* (Robicsek et al., 2006), *Enterobacter* sp. (Robicsek et al., 2006; Cattoir et al., 2007), *Citrobacter freundii* (Cattoir et al., 2007) *Klebsiella pneumoniae* (Robicsek et al., 2006), *Salmonella T*yphi (Dimitrov et al., 2010; Baker et al., 2013), *Salmonella enterica* (Yanagi et al., 2009), *Shigella flexneri* (Hata et al., 2005), and *Vibrio cholerae* (Ismail et al., 2011). There are multiple mechanisms by which these species acquire resistance to fluoroquinolones. Resistance was initially recognized to be due to point mutations in quinolone resistance determining regions (QRDRs) of the *gyrA* and *parC* quinolone target genes and may also be attributable to other chromosomal genes that promote quinolone efflux. Secondly, plasmid mediated quinolone resistance (PMQR) can be mediated through QRDR-protective *qnr* alleles, the ciprofloxacin-acetylating *aac(6)-Ib-cr* allele or quinolone-efflux genes *qepA* and *oqxAB* (Redgrave et al., 2014; Blair et al., 2015). In general fluoroquinolone-resistant strains could have multiple genetic bases for resistance. Some accumulate multiple QRDR mutations and others carry one or more PMQR genes with one or more QRDR mutations. Sequential transition of a strain to higher level resistance is generally attributable to multiple but independent genetic events. Selective pressure from widespread fluroquinolone use appears to have allowed clones that have independently acquired a combination of natural mutation and recombination events to expand internationally (Hooper, 2003; Strahilevitz et al., 2009; Redgrave et al., 2014).

Fluoroquinolone use is an important selector of evolutionary successful resistant lineages but unlikely to be the only one (Baker et al., 2013). More research is needed to promote in-depth understanding of the drivers of emergence and persistence of resistant lineages. Bacterial clones are, by definition, descended from a common ancestor. Evolution within clones and horizontal gene transfer among them subsequently leads to diversification so that organisms of clonal origin may not always be easily discernible and clonal expansion could be difficult to distinguish from evolutionary convergence. Clone descriptions vary and only a subset of resistance mechanisms receive sufficient focus.

Examples of well-known clonal lineages include lineages of *E. coli* ST131, the fluoroquinolone insensitive and multi-drug resistant (MDR) *Salmonella* Typhimurium DT104, and the ciprofloxacin-resistant *S. enterica* serotype Kentucky ST198-X1-SGI1 clone. These clonal groups are marked by rapid and extensive dissemination and have been extensively tracked in many parts of the world (Threlfall, 2000; Le et al., 2013; Petty et al., 2014). However, “worldwide” or “global” studies of these and other clones frequently under-represent African isolates, and in some cases omit them altogether, pointing to the need for a systematic review of published literature on clones in Africa.

The very definition of a “resistant clone” can vary among studies causing problems in understanding the true scope of clonal emergence and subsequent expansion. Genetic studies, preferably sequence-based, are the gold standard for defining clones. Such studies include phylogenetic studies of resistant bacteria that have been characterized via multilocus sequence typing/analysis or whole genome sequencing (WGS) (Roumagnac et al., 2006; Dahiya et al., 2013; Wong et al., 2015). Sequence analysis of resistance genes or plasmids in the context of well-characterized strains can also indicate clonality but, in the absence of host background sequence, cannot robustly differentiate genetically distinct clones. Other studies characterize resistant clones by phenotypic methods such as serogrouping and resistant profiles or pulse-field gel electrophoresis (PFGE; Ke et al., 2014), which looks at genetic profile based on restriction digestion sites. Africa does have an international network to compare PFGE profiles across countries (http://www.pulsenetinternational.org/networks/africa/). However, it is known that PFGE profiles of a single clone may not be identical or chromosomal rearrangements may produce significantly different profiles within a clone (Ismail et al., 2011; Price et al., 2013). Mobile elements can be acquired in different lineages and identical phenotypic characteristics do not necessary imply genetic relatedness (Chattaway et al., 2014).

A clone definition is as good as the discovery methodology. Thus clones are often redefined when more and better information become available. Phenotypic traits—such as serogrouping, virulence, or biochemical profiling that can be acquired convergently in different lineages—cannot be accurately used to track clonal expansion. This has important implications for much of the literature from Africa where sequence-based studies are few. However, the so-called next generation and indeed third-generation sequencing technologies offer an affordable solution to sequence-based clone identification that is increasingly adopted in African studies with great potential for the future. Our review of global fluoroquinolone resistance and clonal expansion in Africa included genetic methodologies such as WGS, detection, or sequencing of fluoroquinolone resistance genes with well characterized strains as the eligible criteria.

## Systematic search methods

This systematic review was conducted in accordance with the preferred reporting items of systematic reviews and meta-analyses (PRISMA) guidelines (Moher et al., 2009). Relevant English articles available in the Medline (Pubmed) and African Journals Online (AJOL) databases were retrieved by two authors using predefined search terms (Table S1). The literature search was conducted until October 2015.

**Figure 1:**
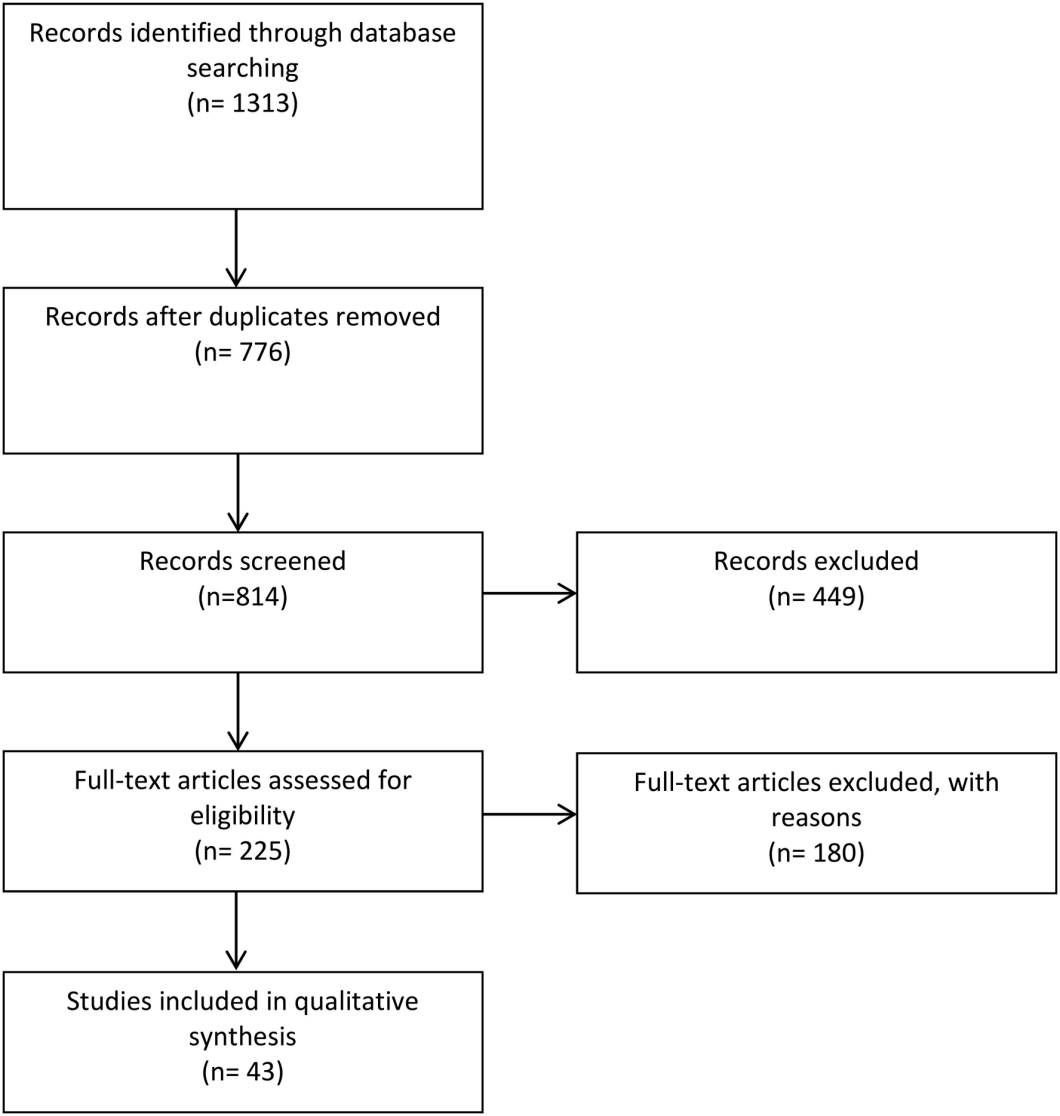
**Summary of study selection process**.

Figure 1 summarizes the study selection process. All duplicate articles were removed. Fluoroquinolone resistant organisms are defined as those having minimum inhibitory concentrations above the CLSI breakpoints (2 μg/ml for ciprofloxacin) or zone sizes below the CLSI breakpoints in standardized disk tests. Strains with MICs above the susceptible breakpoint (0.06 μg/ml and 1 μg/ml for ciprofloxacin in *Salmonella* and other enterics respectively) are described as fluoroquinolone non-susceptible in this review. The eligibility of published reports in this review was based primarily on polymerase chain reaction (PCR) or sequence-based detection of fluoroquinolone resistance genes and the use of at least one molecular tool for assessing clonality of the enteric pathogen including multi-locus sequencing typing (MLST), multi-locus sequencing analysis (MLSA), clone-specific PCR, or PFGE, subsequent sequencing of fluoroquinolone resistance genes, PCR amplicons, and WGS of the enteric organism. Studies where only phenotypic resistance screening methods were used were excluded.

## Results

### Literature search and characteristics of the studies included in the systematic review

The systematic search of the two electronic databases yielded 1313 articles (Figure 1). After the removal of duplicate studies and assessments of titles and abstracts, 776 full-text articles were screened of which 43 studies were considered eligible for the qualitative analysis according to our inclusion criteria (three articles were not included due to inaccessibility). The majority of the articles retrieved were single center studies. Altogether, eligible studies were identified from 17 African countries, less than half of those south of the Sahara. Furthermore, 25 of these studies—almost two-thirds—were performed in just four countries: CAR, Kenya, Nigeria, and South Africa.

The search retrieved articles focused on *V. cholerae, E. coli*, and other Enterobacteriaceae and *Campylobacter* spp. but overall, published work on fluoroquinolone-resistant enteric clones in Africa is predominantly focused on *Salmonella* and whole genome sequence data-based studies are almost entirely limited to this genus. One possibility is that the identification of hypervirulent invasive non-typhoidal *Salmonella* lineages on the continent (Okoro et al., 2012) is fueling this research. However, our reading of the literature appears to point to the enhanced focus on the molecular epidemiology of *Salmonella* in East and Southern Africa as being responsible for identifying this clonal expansion problem in the first place and it is probable that expanded research on other pathogens will uncover other local clones.

Many studies seeking fluoroquinolone-resistant strains found strains with intermediate susceptibility and/or strains that were resistant to the quinolones but not the fluoroquinolones (Table S2). As resistance to the fluoroquinolones typically evolves in a stepwise fashion, the wide prevalence of these intermediate strains is worrisome. While there are very few studies reporting fluoroquinolone-resistant *E. coli* clones, reports are increasingly using sequence-based methods to identify clones, specifically, MLST. The predominance of MLST has been helpful in placing the findings from African countries into global context and demonstrates the extension of knowledge that can come from using sequence-based tools in small studies (**Tables 2, 3**). There are two *E. coli* MLST schemes in the literature and whilst the Lacher et al. scheme (Lacher et al., 2007) has been used in African studies (Feglo et al., 2013), the more widely used (Wirth et al., 2006) scheme is more frequently applied, making comparisons among these many small studies possible. Where MLST was not applied *per se*, PCR markers for MLST-defined ST131 clades were used to identify members of those clones (Peirano et al., 2011, 2014; Albrechtova et al., 2012; Gqunta and Govender, 2015). Methodology of this type could be developed for and applied to other clones that are later found to be of interest in Africa (Doumith et al., 2015). A few African studies, generally older ones, have used PCR-based techniques and PFGE (Lavollay et al., 2006; Kariuki et al., 2007; Peirano et al., 2011). While these studies are the principal sources of information on distribution of FQREC soon after these strains evolved in Africa, it is difficult to connect the clones identified therein to those reported more recently.

Data from *Salmonella* are predominantly available from Eastern and Southern Africa, and there are two multicountry studies focused on Salmonella Kentucky ST198 (Le et al., 2011, 2013). *V. cholerae* studies reported from outbreaks in Western (Nigeria, Cameroon), Eastern (Kenya), and Southern Africa with one pair of studies focused on the same outbreak on either side of the Nigeria-Cameroon border (Quilici et al., 2010; Marin et al., 2013). Most *E. coli* data studied in this review came from single country studies, limited to CAR (Janatova et al., 2014), Ghana (Namboodiri et al., 2011; Feglo et al., 2013) Kenya (Kariuki et al., 2007; Albrechtova et al., 2012), Nigeria (Lamikanra et al., 2011; Aibinu et al., 2012a), and South Africa (Gqunta and Govender, 2015). Intercontinental studies generally included isolates from only one sub-Saharan African country (CAR or South Africa) and other non-African countries (Lavollay et al., 2006; Peirano et al., 2011, 2014). Therefore there are no data comparing FQREC isolates from different countries in sub-Saharan Africa. Additionally, multicountry or “global” studies are typically including only one country from within sub-Saharan Africa, even though it is well known that biological diversity on the African continent exceeds that in most other parts of the world.

### Vibrio cholerae

Cholera, the pandemic diarrheal disease caused by toxigenic *V. cholerae* strains, has become increasingly problematic across Africa in the last two decades but fluoroquinolone resistance has only been recently studied (Scrascia et al., 2006, 2009; Mintz and Guerrant, 2009), Quilici et al. (2010) described the microbiology of a cholera outbreak that spanned the borders of Nigeria and Cameroon in 2009 and (Quilici et al., 2010) was due to a single *V. cholerae* O1 Ogawa El-tor clone. The *ctxB* DNA sequence from this clone was similar (only 3 SNPs) to a strain identified in India in 2007 but different from variants isolated in southern Africa, indicating a possible transmission from Asia to Africa. This clone was resistant to nalidixic acid and MICs to ciprofloxacin were 0.25–0.5 μg/ml, placing them in the susceptible category (based on general enterobacterial breakpoints), although these MICs approach the reduced susceptibility breakpoint of 1 μg/ml. Strains belonging to this clone contained mutations in the QRDR of *gyrA* (Ser83-Ile) and *parC* (Ser85-Leu) but no mutations in *gyrB* or *parE*. A more comprehensive Nigeria study looking at *V. cholerae* O1 strains isolated in the early 1970's and comparing them with strains of *V. cholerae* O1 and non-O139 strains isolated in 2009–2010 indicated that *gyrA* (Ser83-Ile) and *parC* (Ser85-Leu) mutations were not present in the 1970's or in non-O1 clinical isolates (Marin et al., 2013). Interestingly, MLVA analysis clustered the 1970's O1 El-tor strain strains with epidemic O1 El-tor strains but the strains had different *ctxB* types and vibrio 17th pandemic island (VSP-II) profiles indicating that they do not represent one clone (Marin et al., 2013). This can be explained by the housekeeping genes used in this analysis (*pyrH, recA*, and *mdh*) being highly conserved in *V. cholerae* serogroup strains. Approaches using whole genome sequence may be required to further define the inter-relatedness of these O1 strains.

The most in-depth assessment of fluoroquinolone resistant mechanisms was a South African study focused on a *V. cholerae* O1 Ogawa El-tor clone outbreak (Ismail et al., 2011). A selection of strains was assessed for the presence of plasmid-mediated quinolone resistance (PMQR) genes (*qnrA, qnrB, qnrS, qnrC*, and *qepA)* as well as *Vibrio* quinolone resistance determinant (*qnrVC3*). Nalidixic acid resistance was investigated in the presence and absence of two efflux pump inhibitors and QRDR mutations were identified by sequencing. The South Africa strains were negative for any PMQR genes and there was no evidence of active efflux conferring quinolone resistance. Nalidixic acid resistance was attributable to *gyrA* (Ser83-Ile) and *parC* (Ser85-Leu) mutations (Ismail et al., 2011). All of the recent outbreak strains described in these three studies were *V. cholerae* O1 El-tor (two studies also indicated serogroup Ogawa) which showed resistance to nalidixic acid but only a reduced susceptibility to ciprofloxacin (Table 1). It is highly likely that these strains are the same clone and that the same QRDR mutations are responsible for fluoroquinolone resistance. High levels of resistance to nalidixic acid or reduced susceptibility to ciprofloxacin in likely similar *V. cholerae* O1 clones causing epidemics in DRC and Kenya have also been shown (Mercy et al., 2014; Miwanda et al., 2015; Table 1), but a lack of standard methodology for clonal analysis prevents an understanding of clonal spread across Africa. Although ciprofloxacin has only a reduced susceptibility in these strains and continues to be used for cholera management, if additional mutations occur in these circulating clones, resistance to ciprofloxacin may develop.

**Table 1:** Characterization of eligible articles that sought fluoroquinolone resistant clones in humans and animals^**^.

**Country**	**Organism**	No. resistant^**^/No total	**ID method**	**Study period**	**Study population**	**Genotyping tools**						**Resistance testing**				**References**
						**WGS**	**MLST**	**ERIC-PCR**	**PFGE**	**Rep PCR**	Other^*^	**PMQR**	**QRDR**	**Efflux pump up- regulation**	***aac(6′)-Ib-cr***	
Angola	*E. coli*	9/19	B, S	2009	Stray dogs	–	–	–	√	–	–	√	–	–	–	Albrechtova et al., 2014a
Cameroon	*V. cholerae* O1 Ogawa El-tor	0/9	B, S, M	2009	Outbreak	–	–	–	–	–	–	–	√	–	–	Quilici et al., 2010
Central African Republic	*Enterobacteriaceae*	33/121	B, S	2011	Human/wildlife	–	–	–	√		–	√	–	–		Janatova et al., 2014
	*Enterobacteriaceae*	65/65	B	2011–2012	Clinical isolates (surgical site infections)	–	√	√	–	√	–	√	–	–	√	Rafai et al., 2015
	*E. coli*	10/121	B, M	2011	Gorillas and other wildlife	–	√	–	–	–	–	√	–	–	√	Janatova et al., 2014
	*E. coli*	10/10	B, M	2000–2004	Hospital clinical isolates	–	–	–	√	√	–	–	–	–	√	Lavollay et al., 2006
	*S*. Typhi	0/19	B, S	1916–2004	Various	√	–	–	–	–	–	–	–	–	–	Holt et al., 2008
Cote d'Ivoire	*E. coli, Enterobactericeae*	44/101	B, S	2012	Human, dog, wild life	–	–	–	√	–	–	√	–	–	√	Albrechtova et al., 2014b
DRC	*Salmonella* (NTS)	0/233	B, S	2007–2011	Community and Clinical isolates	–	–	–	√	–	–	√	√	–	–	Lunguya et al., 2013
	*V. cholerae*	0/1093	B, S	1997–2012	Clinical Isolates	√	–	–	–	–	MLVA	–	–	–	–	Miwanda et al., 2015
Ghana	*E. coli*	23/293	B, M	2006–2008	Clinical isolates from tertiary care hospitals	–	√	–	–	–	–	√	√	–	–	Namboodiri et al., 2011
	*E. coli*	28/29	B, M	2008–2009	Clinical isolates: Blood, urine, sputum and wound	–	√	–	–	–	–	–	–	–	–	Feglo et al., 2013
Ethiopia	*Salmonella*	19/98	B, S	2000–2005	Animal/Animal products	–	–	–	√	–	–	–	–	–	–	Molla et al., 2007
Kenya	*E. coli*	66/289	B, M	2009	Dogs (mainly), cats and humans	–	–	–	√	–	–	√	–	–	√	Albrechtova et al., 2012
	*E. coli*	12/17	B, M	2004–2005	Clinical isolates: Urine	–	–	–	√	–	–	√	–	–	√	Kariuki et al., 2007
	*V. cholerae*	1/76	B, S	2007–2010	Clinical Isolates	–	–	–	√	–	Ribo-typing	–	–	–	–	Mercy et al., 2014
	*S. Kentucky*	3/3	B, S	2000–2005	Clinical Isolates	–	–	–	√		–	√	√	–	–	Weill et al., 2006
	*S*. Typhi	0/94	B, S	1988–2008	Clinical isolates	√	–	–	–	–	–	–	–	–	–	Kariuki et al., 2010
	*S*. Typhi	0/102	B, S	2001–2002	Clinical isolates	–	–	–	√	–	–	–	√	–	–	Kariuki et al., 2004
	*S*. Typhi	NS/55	NS	2001–2009	Clinical isolates	√	–	–	–	–	–	–	–	–	–	Wong et al., 2015
Malawi	*S*. Typhi	0/112	B, BC	1998–2013	Clinical isolates (blood)	√	√	–	–	–	–	–	–	–	–	Feasey et al., 2015
	*S*. Typhi	NS/112	NS	2004–2013	Clinical isolates	√	–	–	–	–	–	–	–	–	–	Wong et al., 2015
Nigeria	*E. coli*	6/162	B, S	2006	Poultry/Pigs	–	–	√	–	–	–	√	–	–	√	Fortini et al., 2011
	*E. coli*	57/114	B, M	2010–2011	Human clinical isolates, bovine isolates from farms	–	√	–	–	√	–	√	–	–	√	Inwezerua et al., 2014
	*E. coli*	26/32	B, M	2011	Humans (pregnant women)	–	√	√	–	–	–	√	–	–	√	Fortini et al., 2015
	*E. coli*	9/109	B, M	2008–2009	Clinical isolates from tertiary care hospitals	–	ST131 and ST10	√	–	–	–	√	–	–	√	Aibinu et al., 2012a
	*E. coli*	21/121	B, M	2005	Healthy volunteers	–	√	–	–	–	*fliC* -RFLP	–	√	√	–	Lamikanra et al., 2011
	*Salmonella* (NTS)	11/149	B, S	2009–2011	Human/poutry/cattle/fish/vegetable	–	–	–	–	–	–	√	–	–	–	Raufu et al., 2013
	*Salmonella* (NTS)	2/229	B, S	2010–2011	Pigs	–	–	–	–	–	–	√	–	–	–	Fashae and Hendriksen, 2014
	*S*. Hiduddify	24/130	B, S	2001	Chickens/poultry meat	–	–	–	√	–	–	–	–	–	–	Raufu et al., 2009
	*S*. Kentucky	197/197	B, S	1959–2008	Human/Poultry/sea food/river/environment	–	√	–	√	–	–	√	√	–	√	Le et al., 2011
	*S*. Kentucky	55/55	B, S	2007–2011	Poultry/poultry sources	–	–	–	√	–	–	–	–	–	–	Raufu et al., 2014
	*S*. Kentucky	54/70	B, S	1937–2013	Humans/animals/environment	–	√	–	√	–	–	√	√	–	√	Le et al., 2013
	*V. cholerae* O1 and non O1	0/20	B, S, M	1971 –2010	Outbreak, historical, environmental	–	–	–	√	–	MLSA	–	√	–	–	Marin et al., 2013
	*V. cholerae* O1 Ogawa El-tor	0/10	B, S, M	2009	Outbreak	–	–	–	–	–	–	–	√	–	–	Quilici et al., 2010
	*E. coli*	NS/12	B, M	2000–2010	Clinical isolates	–	√	–	√	–	Lineage PCR	–	√	–	√	Peirano et al., 2014
	*E. coli*	0/22	B, M	2008–2009	Clinical isolates (Urine and pus)	–	–	–	√	–	–	√	–	–	√	Peirano et al., 2011
Senegal	*C. coli*	14/36	B, M	2000–2002	Chicken	–	√	–	–	–	–	–	√	–	–	Kinana et al., 2007
	*Salmonella (*NTS) *S. Typhi*	4/1623	B, S, M	1999–2009	Clinical isolates	–	–	–	–	–	–	√	√	–	–	Harrois et al., 2014
	*C. jejuni*	19/46	B, M	2000–2002	Chicken	–	√	–	–	–	–	–	√	–	–	Kinana et al., 2006
	*C. jejuni, C. coli*	81/181	B, M	2000–2003	Poultry, Human	–	–	–	√	–	–	–	√	–	–	Cardinale et al., 2006
Sierra Leone	*V. cholerae* O1	0/15	B, M, S	2012	Clinical isolates	–	–	–	√	–	–	–	–	–	–	Mahmud et al., 2014
South Africa	*E. coli*	14/21	B, M	2012–2013	Clinical isolates	–	√	–	√	–	–	√	–	–	√	Gqunta and Govender, 2015
	*S*. Typhi	1/1	B, S	2010	Clinical isolate	–	–	–	√	–	–	√	√	–	–	Keddy et al., 2010
	*S*. Typhi	0/510	B, S	2003–2007	Clinical isolates	–	–	–	√	–	MLVA	√	√	–	–	Smith et al., 2010
	*S*. Typhi	NS/41	NS	2004–2012	Clinical isolates	√	–	–	–	–	–	–	–	–	–	Wong et al., 2015
	*S*. Kentucky	1/1	B, S	2000–2005	Clinical Isolates	–	–	–	√	–	–	√	√	–	–	Weill et al., 2006
	*V. cholerae* O1 Ogawa El-tor	0/31	B, S, M	2008	Outbreak	–	–	–	√	–	–		–	–	–	Ismail et al., 2011
Tanzania	*S*. Typhi	NS/52	NS	2006–2010	Clinical isolates	√	–	–	–	–	–	–	–	–	–	Wong et al., 2015
	*S*. Kentucky	3/3	B, S	2000–2005	Clinical Isolates	–	–	–	√		–	√	√	–	–	Weill et al., 2006

*KEY: ID method: B, Biochemistry; S, Serotyping; M, detection of target genes; BC, Blood culture; √, test was conducted; –, test not conducted; NS, Not stated (data not included in manuscript)*.

* *Other, where specific genotypic methods are used to identify clones of that enteric pathogen*.

** *In some cases where fluoroquinolone-resistant clones were sought but only quinolone resistant or non-susceptible isolates were identified. Details of these studies are provided in Table S2*.

*WGS, Whole genome sequencing; MLST, Multi-locus sequence typing; MLSA, Multi-locus sequencing analysis; MLVA, multi-locus variable-number tandem repeat analyses; PFGE, pulse field gel electrophoresis; ERIC-PCR, Enterobacterial Repetitive Intergenic Consensus – PCR; REP-PCR, repetitive element palindromic PCR; RFLP, restriction fragment length polymorphism; NTS, Nontyphoidal Salmonella, Clinical Isolates – from humans; PMQR, plasmid mediated quinolone resistance (including qnrA, qnrB, qnrS, qnrC, qepA, and qnrVC3); QRDR, quinolone resistance-determining region [including sequencing the QRDR of DNA gyrase (gyrA, gryB) and topoisomerase IV (parC, parE) genes]; efflux, efflux up regulation testing; aac(6′)-Ib-cr, aminoglycoside acetyltransferace associated with ciprofloxacin resistance; DRC, Democratic Republic of the Congo*.

### Campylobacter

*Campylobacter jejuni* is a leading enteric bacterial pathogen in developing countries particularly among children below the age of 5 years. In spite of numerous reports of *C. jejuni* as well as its resistance to fluoroquinolones (Coker et al., 2002) data on clones of the organisms causing human infections in Africa are non-existent. Characterization of 46 isolates from chickens in Senegal by MLST revealed seven clonal complexes which have all been associated with human disease elsewhere (Kinana et al., 2006). Clonal complex 353 was the most common and Thr-86-Ile substitution in *gyrA* was the major mechanism of quinolone resistance. Rather than diffusion of a single or a few clones, acquisition of resistance appears to be occurring in different lineages in response to selective pressure. Similarly, another study from Senegal, strain typing of 99 chicken and 10 human isolates of *C. jejuni* by PFGE that uncovered common patterns among strains circulating in human and poultry, though were no particular predominant pulsotypes (Cardinale et al., 2006). Altogether, although the data are few, there is yet to be robust evidence for expanded fluoroquinolone resistant *Campylobacter* clones.

### Enteric and commensal *Escherichia coli* and *Shigella*

Diarrhea was the third leading cause of death in children under five in sub-Saharan Africa in 2013 (after malaria and pneumonia), accounting for 12% of the estimated 3.6 million deaths (Liu et al., 2015). In sub-Saharan Africa and south Asia, most attributable cases of moderate-to-severe childhood diarrhea in under-fives are due to the enteric bacteria *E. coli* and *Shigella* (Kotloff et al., 2013). While there was a report of a single quinolone-resistant *Shigella* isolate from Zaire in 1985 (Frost et al., 1985) and *Shigella* epidemics caused by quinolone resistant bacteria in the last century (Goma Epidemiology Group, 1995; Karas and Pillay, 1996; Vlieghe et al., 2009) we did not identify reports of fluoroquinolone-resistant clones of enteric *E. coli* (FQREC) in the literature before 2000. Available reports appear to suggest that most isolates were fluoroquinolone susceptible until the end of the 1990s (Wistrom et al., 1987; Felmingham and Robbins, 1992; Thornton et al., 1992; Bourgeois et al., 1993; Oyofo et al., 1997; Decousser et al., 1999; Vila et al., 1999; Ahmed et al., 2000; Okeke et al., 2000a,b; Jiang et al., 2002; Orogade and Akuse, 2004). Our findings are corroborated by other recent systematic reviews (Vlieghe et al., 2009; Leopold et al., 2014).

Reports of fluoroquinolone-resistant *E. coli* from cases of diarrhea and commensal reservoirs in Africa are limited to the current millennium (Aibinu et al., 2004; Lamikanra et al., 2011; Namboodiri et al., 2011; Fortini et al., 2015). One study that examined human commensal isolates from Ghana (2006–2008) found a wide range of STs associated with fluoroquinolone resistance (Namboodiri et al., 2011). However, strains belonging to the multilocus sequence type (ST) complex (specifically STs 10, 227, and 617) and the ST 156 sequence type complex (STs 156 and 1473) were detected 8 and 3 times respectively representing half of the total number (22) of FQREC detected in the study. An additional five ST10 isolates were resistant to nalidixic acid and overall, ST10 strains were computed to be overrepresented among quinolone-resistant *E. coli* (QREC) from Ghana (*p* = 0.02, Fishers exact test; Namboodiri et al., 2011). Five ST10 QREC (including four FQREC) harbored *qnrS1*, whilst other strains carried *qepA* or *qnrB2* genes. The ST156 strains harbored three or more QRDR mutations, in contrast to most other strains that had 0–2 and one of the ST156 isolates also carried *qepA*. Interestingly, multiple *qepA*-bearing FQREC ST156 strains have also been reported as human commensals in the Central African Republic and Nigeria (Table 2; Janatova et al., 2014; Fortini et al., 2015). These and other lineages could be more broadly disseminated but remain unflagged because data are insufficient or unavailable. For example, *qnrS1*-bearing clones that have not been multilocus sequence typed (clonality was inferred by ERIC-PCR) have also been isolated from humans and pigs in Nigeria (Fortini et al., 2011, 2015).

**Table 2:** **Multilocus sequence types of quinolone resistant commensals reported from potential reservoirs in sub-Saharan Africa**.

**City, Country**	**Host organism**	**Year**	**Number of independent isolates**	**ST**	**Quinolone resistance mechanisms**	**References**
Accra, Ghana	Human	2006	2	*E. coli* (ST101)	gyrA (S83L, D87N); *qnrB1*	Namboodiri et al., 2011
Accra, Ghana	Human	2006	1	*E. coli* (ST617)	gyrA (S83L, D87N); parC (S80I); *qnrS1*	Namboodiri et al., 2011
Accra, Ghana	Human	2006	2	*E. coli* (ST648)	gyrA (S83L, D87N); parC (S80I)	Namboodiri et al., 2011
Accra, Ghana	Human	2006	1	*E. coli* (ST455)	gyrA (S83L, D87N); parC (S80I, N105S)	Namboodiri et al., 2011
Accra, Ghana	Human	2006, 2007	2	*E. coli* (ST156)	gyrA (S83L, D87N); parC (S80I, E84K), qepA	Namboodiri et al., 2011
Accra, Ghana	Human	2007	1	*E. coli* (ST1304)	None detected	Namboodiri et al., 2011
Accra, Ghana	Human	2007	1	*E. coli* (ST450)	gyrA (S83L, D87N)	Namboodiri et al., 2011
Accra, Ghana	Human	2007, 2008	5	*E. coli* (ST10)	gyrA (S83L, D87N); parC (S80I), qepA, qnrS1	Namboodiri et al., 2011
Accra, Ghana	Human	2007	1	*E. coli* (ST354)	gyrA (S83L, D87N); parC (S80I, E84G), qnrB	Namboodiri et al., 2011
Accra, Ghana	Human	2008	1	*E. coli* (ST1473)	None detected	Namboodiri et al., 2011
Accra, Ghana	Human	2008	1	*E. coli* (ST1496)	None detected	Namboodiri et al., 2011
Accra, Ghana	Human	2008	2	*E. coli* (ST227)	gyrA (S83A), qnrS1	Namboodiri et al., 2011
Accra, Ghana	Human	2008	1	*E. coli* (ST410)	gyrA (S83L, D87N); parC (S80I)	Namboodiri et al., 2011
Accra, Ghana	Human	2008	1	*E. coli* (ST1468)	gyrA (S83L, D87N); parC (S80I)	Namboodiri et al., 2011
Accra, Ghana	Human	2008	1	*E. coli* (ST131)	gyrA (S83L, D87N); parC (S80I, E84V)	Namboodiri et al., 2011
Accra, Ghana	Human	2008	1	*E. coli* (ST1470)	*gyrA* (S83L, D87N); *parC* (S80I, A108V)	Namboodiri et al., 2011
Dzanga, CAR	African buffalo	2011	1	*K. pneumoniae* (ST1208)	*oqxA*	Janatova et al., 2014
Dzanga, CAR	African buffalo	2011	1	*K. pneumoniae* (ST1209)	*oqxA*	Janatova et al., 2014
Dzanga, CAR	Peter's Dunker	2011	1	*K. pneumoniae* (ST1210)	*oqxA*	Janatova et al., 2014
Dzanga, CAR	Human	2011	4	*E. coli* (ST3476)	*qnrS1*	Janatova et al., 2014
Dzanga, CAR	Human	2011	3	*E. coli* (ST156)	*qepA*	Janatova et al., 2014
Dzanga, CAR	Human	2011	1	*E. coli* (ST69)	*oqxA*	Janatova et al., 2014
Dzanga, CAR	Human	2011	1	*E. coli* (ST218)	*qnrB1*	Janatova et al., 2014
Dzanga, CAR	Gorilla	2011	1	*E. coli* (ST424)	*qepA*	Janatova et al., 2014
Northern Kenya	Dogs	2012	3	*E. coli* (ST131^*^)	*aac(6')-Ib-cr*	Janatova et al., 2014
Ile-Ife, Nigeria	Human	2005	9	*E. coli* (ST10 complex)	*gyrA* (S83L), efflux	Lamikanra et al., 2011
Ile-Ife, Nigeria	Human	2005	2	*E. coli* (ST452)	efflux	Lamikanra et al., 2011
Ile-Ife, Nigeria	Human	2005	2	*E. coli* (ST517)	efflux	Lamikanra et al., 2011
Ile-Ife, Nigeria	Human	2005	1	*E. coli* (ST494)	efflux	Lamikanra et al., 2011
Ile-Ife, Nigeria	Human	2005	1	*E. coli* (ST156)	efflux	Lamikanra et al., 2011
Ile-Ife, Nigeria	Human	2005	1	*E. coli* (ST521)	efflux	Lamikanra et al., 2011
Ile-Ife, Nigeria	Human	2005	1	*E. coli* (ST448)	*gyrA* (S83L, D87N); *parC* (S80I), efflux	Lamikanra et al., 2011
Ile-Ife, Nigeria	Human	2005	1	*E. coli* (ST503)	efflux	Lamikanra et al., 2011
Ile-Ife, Nigeria	Human	2005	1	*E. coli* (ST504)	*gyrA* (S83L), efflux	Lamikanra et al., 2011
Ibadan, Nigeria	Human	2011	1	*E. coli* (ST10)	*qnrS1, aac(6')-Ib-cr*	Fortini et al., 2015
Ibadan, Nigeria	Human	2011	1	*E. coli* (ST156)	*qepA1*	Fortini et al., 2015
Ibadan, Nigeria	Human	2011	1	*E. coli* (ST3147)	*qnrS1*	Fortini et al., 2015
Senegal	Poultry	2000–2002	11	*C. jejuni* (ST1036)	*gyrA* (H81, S119, T86I, G110)	Kinana et al., 2006
Senegal	Poultry	2000–2002	3	*C. jejuni* (ST1040)	*gyrA* (H81, S119, T86I)	Kinana et al., 2006
Senegal	Poultry	2000–2002	5	*C. jejuni* (ST1039)	*gyrA* (H81, S119)	Kinana et al., 2006
Senegal	Poultry	2000–2002	3	*C. jejuni* (ST1041)	*gyrA* (H81, S119, T86A, G110)	Kinana et al., 2006
Senegal	Poultry	2000–2002	1	*C. jejuni* (ST1081)	None detected	Kinana et al., 2006
Senegal	Poultry	2000–2002	1	*C. jejuni* (ST660)	None detected	Kinana et al., 2006
Senegal	Poultry	2000–2002	1	*C. jejuni* (ST22)	None detected	Kinana et al., 2006
Senegal	Poultry	2000–2002	1	*C. jejuni* (ST1037)	*gyrA* (H81, S119, T86I)	Kinana et al., 2006
Senegal	Poultry	2000–2002	3	*C. jejuni* (ST1038)	*gyrA* (H81, S119)	Kinana et al., 2006
Senegal	Poultry	2000–2002	4	*C. jejuni* (ST52)	*gyrA* (H81, S119)	Kinana et al., 2006
Senegal	Poultry	2000–2002	1	*C. jejuni* (ST824)	*gyrA* (H81, S119)	Kinana et al., 2006
Senegal	Poultry	2000–2002	1	*C. jejuni* (ST1359)	*gyrA* (H81, S119)	Kinana et al., 2006
Senegal	Poultry	2000–2002	1	*C. jejuni* (ST1211)	*gyrA* (H81, S119)	Kinana et al., 2006
Senegal	Poultry	2000–2002	1	*C. jejuni* (ST1370)	*gyrA* (H81, S119)	Kinana et al., 2006
Senegal	Poultry	2000–2002	1	*C. jejuni* (ST1358)	*gyrA* (H81, S119)	Kinana et al., 2006
Senegal	Poultry	2000–2002	8	*C. jejuni* (ST1035)	*gyrA* (H81, S119, G110, T86A, T86I)	Kinana et al., 2006

* *Determined by marker-based PCR not MLST*.

### Invasive *Escherichia coli*

Fluoroquinolone-resistant *E. coli* are a rising cause of invasive infections in Africa. The majority of reports of fluoroquinolone-resistant enterobacterial lineages only seek lineage information among ESBL-producers (Table 3; Aibinu et al., 2012a; Isendahl et al., 2012; Breurec et al., 2013; Gqunta and Govender, 2015; Rafai et al., 2015) so that the true prevalence of fluoroquinolone-resistant strains is unknown as is the existence of ESBL-negative fluoroquinolone-resistant lineages of importance. Interestingly, while the data are few and biased toward ESBL-positive lineages, there is some overlap between STs of FQREC recovered from clinical infections and those found in the commensal reservoir from humans and wild mammals (Tables 2, 3).

**Table 3:** **Multilocus sequence types of fluoroquinolone-resistant ***E. coli*** clinical isolates reported from sub-Saharan Africa**.

**City, Country**	**Isolation source**	**Year**	**Number of independent isolates**	**ST**	**Reported quinolone resistance mechanisms**	**Other resistances**	**References**
Kumasi, Ghana	Blood, Urine, sputum and wounds	2008–2009	28	88 Whittam scheme (Lacher et al., 2007)	None reported	ESBLs, gentamicin, trimethoprim-sulphamethoxazole, chloramphenicol	Feglo et al., 2013
Bangui, CAR	Surgical site infections	2011–2012	12	10	*qnrB,qnrS, aac(6')-Ib-cr*	ESBLs	Rafai et al., 2015
Bangui, CAR	Surgical site infections	2011–2012	1	146	*qnrB, qnrS, aac(6')-Ib-cr*	ESBLs	Rafai et al., 2015
Bangui, CAR	Surgical site infections	2011–2012	12	131	*qnrB, qnrS, aac(6')-Ib-cr*	ESBLs	Rafai et al., 2015
Bangui, CAR	Surgical site infections	2011–2012	5	156	*qnrB, qnrS, aac(6')-Ib-cr*	ESBLs	Rafai et al., 2015
Bangui, CAR	Surgical site infections	2011–2012	1	354	*qnrS, aac(6')-Ib-cr*	ESBLs	Rafai et al., 2015
Bangui, CAR	Surgical site infections	2011–2012	2	405	*qnrB, aac(6')-Ib-cr*	ESBLs	Rafai et al., 2015
Cape Town, South Africa	Urine and Pus	2008–2009	5	131^*^	*aac(6')-Ib-cr*	ESBLs	Peirano et al., 2011
Ibadan, Nigeria	Urine	2010–2011	1	2695	*qnrB, aac(6')-Ib-cr*	ESBLs	Inwezerua et al., 2014
Ibadan, Nigeria	Vaginal	2010–2011	1	131	*aac(6')-Ib-cr*	ESBLs	Inwezerua et al., 2014
Lagos, Nigeria	Urine	2011	2	131	*qnrB, aac(6')-Ib-cr*		Aibinu et al., 2012b
Lagos, Nigeria	Urine and stool	2011	2	617 (10 complex)	*qnrB, aac(6')-Ib-cr*		Aibinu et al., 2012b
Lagos, Nigeria	Urine and stool	2011	1	295	*aac(6')-Ib-cr*		Aibinu et al., 2012b
Lagos, Nigeria	Urine and stool	2011	1	23	*aac(6')-Ib-cr*		Aibinu et al., 2012b
Lagos, Nigeria	Urine and stool	2011	1	448	*aac(6')-Ib-cr*		Aibinu et al., 2012b
Lagos, Nigeria	Urine and stool	2011	1	501	*aac(6')-Ib-cr*		Aibinu et al., 2012b
Port Elizabeth, South Africa	Clinical	2012–2013	12	131^*^	*qnrB qnrS, aac(6')-Ib-cr*	ESBLs	Gqunta and Govender, 2015

*E. coli STs are defined by the Achtman scheme unless otherwise indicated (Wirth et al., 2006)*.

* *Determined by marker-based PCR not MLST*.

Kariuki et al. examined multiply resistant *E. coli* from urinary tract infections in Kenya (Kariuki et al., 2007). Two PFGE profiles were seen more than once. Strains belonging to these clones had identical *gyrA* sequences, which included Ser83Leu and Asp87Asn mutations in the QRDR regions of this gene. They also carried a plasmid-borne *aac-6(Ib)-cr* gene and some had *qnrA* and *qnrB* genes. Working largely with strains from other areas but including 10 isolates from Bangui, Lavollay et al. found a globally disseminated clone that carried an *aac6-Ib-cr* allele on its CTX-M-15 bearing plasmid and detected two isolates of this clone, as defined by REP-PCR and PFGE, among the Central African Republic isolates (Lavollay et al., 2006).

Available data suggests that pandemic lineages circulate within Africa. For example, fluoroquinolone-resistant strains belonging to the globally disseminated *E. coli* ST131 lineage, which is actually comprised of multiple clones, have rarely been systematically sought but there are sporadic reports that demonstrate their circulation in CAR (Rafai et al., 2015), Nigeria (Aibinu et al., 2012a; Inwezerua et al., 2014), Guinea Bissau (Isendahl et al., 2012), Tanzania (Lupindu et al., 2014), and South Africa (Peirano et al., 2011, 2014; Gqunta and Govender, 2015; Table 3). These data suggest that global dissemination may be dictated by the pandemic potential of the ST131 lineages rather than actual intercontinental transfer of specific clones.

Aibinu et al (Aibinu et al., 2012a) studied 109 *E. coli* isolates obtained from two tertiary care hospitals in Lagos. Of the 14 isolates that were ESBL-producers, three were ST131 *E. coli*. Chromosomal SNPs were not sought but two of the ST131 isolates and six other ESBL-producers (ST 10 complex—two strains, STs 23, 295, 448, and 501) harbored the *aac-(6*′*)-lb-cr* gene (Table 3). These eight strains and the pMQR-negative ST131 strain all carried the ESBL-encoding CTX-M-15 gene. One ST131 strain additionally carried a qnrB1 gene and a ST 617 strain (ST10 complex) carried *qnrA1*. This study demonstrated the presence of ST131, as well as other notorious *E. coli* clones. Many of the STs reported by Aibinu et al. were also found 2005 in the Nigerian study of Lamikanra et al. (2011) (2009 isolates from that study were not sequenced typed) as well as among 2011 isolates from Fortini et al. (2015). Feglo et al. conducted MLST on a subset of *E. coli* isolates from Kumasi Ghana, hypothesizing that they would find ST131 strains among them. Instead, they found that all of 29 isolates evaluated belonged to the pyleonephritis-associated lineage ST88. Thus, regional or internationally disseminated clones have been successful in African settings and that locally successful clones are not necessarily those with the greatest pandemic reputations.

### *Salmonella* Typhi and non-typhoidal *Salmonella*

*Salmonella* causes an estimated three billion infections in humans and animals yearly (Crump et al., 2004; Coburn et al., 2007). This poses significant socio-economic challenges, especially in resource-limited settings. Classically, *Salmonella* can be divided into two species, *S. bongori* and *S. enterica* (Crosa et al., 1973; Schrire et al., 1987; Reeves et al., 1989). *S. enterica* can be further divided into six sub-groups based on carbohydrate utilization, flagellar, and lipopolysaccharide (LPS) structures (Tindall et al., 2005; Coburn et al., 2007; Grimont and Weill, 2007). Currently there are more than, 2550 serovarieties of *S. enterica. S. enteric*a is responsible for most cases of salmonellosis in human and warm-blooded animals (Brenner et al., 2000). Based on the type of disease syndrome and host specificity, *S. enterica* can be broadly divided into two groups (typhoidal and non-typhoidal). “Typhoidal Salmonellae” are generally human-restricted and cause enteric fever; these include *S*. Typhi and *S*. Paratyphi. The non-typhoidal salmonellae (NTS) encompass several serovars with broad hosts including animals and humans such as *S*. Typhimurium, *S*. Enteritidis, *S*. Poona, and *S*. Isangi. (Dougan et al., 2011). In the WHO Africa sub-region, the burden of non-typhoidal gastroenteritis is estimated at 2.5 million cases and 4100 deaths per year (Majowicz et al., 2010) but this estimate is based on few data. Nontyphoidal *Salmonella* are also risk factors for mortality from childhood diarrhea (O'Reilly et al., 2012). NTS serovars are commonly associated with gastrointestinal disease in hosts but can sometimes cause invasive or systemic disease in humans and animals (Dougan et al., 2011; Langridge et al., 2012).

Emerging epidemiological data from across the subcontinent shows that invasive non-typhoidal Salmonella (iNTS) infection has become a major cause of bloodstream infections, overtaking typhoidal enteric fever in many places, especially among malarial and malnourished children and in HIV-positive adults (Reddy et al., 2010). Mortality rates in these vulnerable populations are reported to be as high as 30% in the absence of adequate treatment (Berkley et al., 2005; Reddy et al., 2010). Multidrug resistance of salmonellae to ampicillin, chloramphenicol and trimethoprim-sulphamethoxazole (traditional regimens, cheap, and orally active) is a global phenomenon, presenting particular salmonellosis management challenges in Africa including the epidemic of invasive *S*. Typhimurium ST 313. This clone consists of two closely related lineages that emerged independently ~ 55 and ~37 years ago, in close temporal association with the HIV pandemic (Kingsley et al., 2009; Okoro et al., 2012). The MDR genes of ST 313 are encoded on a Tn*21*-like element borne on a virulence plasmid pSLT-BT. MDR clones of *S*. Typhi have also emerged and spread intercontinentally and globally involving Africa countries like Kenya (Kariuki et al., 2010; Holt et al., 2011). Prevalence rates of MDR as high as 60.4, 80.7, and 94.7% have been reported from Kenya (Kariuki et al., 2010), DRC (Lunguya et al., 2013), and Malawi (Feasey et al., 2010), respectively. Increasing and dissemination of MDR clones of salmonellae warranted the current use of fluoroquinolones and third generation cephalosporins as the drugs of first choice for treatment (Kariuki and Dougan, 2014; Kariuki et al., 2015a). These newer drugs are expensive and pose significant challenges to resource poor settings. The use of these drugs, together with the use of over-the-counter and substandard antibiotics, has now selected resistance to these drugs in salmonellae (Brenner et al., 2000; Parkhill et al., 2001; McClelland et al., 2004; Berkley et al., 2005; Gordon et al., 2008; Holt et al., 2008; Kingsley et al., 2009; Majowicz et al., 2010; Reddy et al., 2010; Dougan et al., 2011; Langridge et al., 2012; Okoro et al., 2012; O'Reilly et al., 2012; Kariuki and Dougan, 2014; Kariuki et al., 2015a) Initial reports of reduced susceptibility and resistance to quinolones in NTS emerged in the mid 2000's and continue to trickle in from many sub-Saharan African countries including Nigeria (Akinyemi et al., 2007), DRC (Lunguya et al., 2013), Tanzania (associated with travel; Weill et al., 2006), Senegal (Harrois et al., 2014), and Ghana (Nielsen et al., 2012). A Mozambican study identified ~5% of isolates as being resistant to nalidixic acid; resistance to nalidixic acid is regarded as a precursor for 2nd and 3rd generation fluoroquinolone resistance (Mandomando et al., 2009). Recent reports have shown that strains of MDR *S*. Typhimurium ST313 primarily associated with bloodstream infections have acquired an additional incHI2 plasmid that confers additional resistance to many β-lactams including ceftriaxone. These pathovariants have been identified in Kenya (Kariuki et al., 2015b) and Malawi (Feasey et al., 2014). Other reports have shown an association with the acquisition of extended-spectrum β lactamases (ESBLs) and decreased susceptibility to fluoroquinolones in Salmonellae, the latter attributable to plasmid-borne *qnr* genes, although mechanism underlying the significance of this association has not been fully explored (Akinyemi et al., 2007, 2011; Harrois et al., 2014).

There are more than 20 million cases of human typhoid fever resulting in as many as 200,000 fatalities worldwide each year (Crump et al., 2004; Arjyal et al., 2011). Reports of outbreaks of MDR Typhi and Paratyphi emerged in the early 1990's in endemic countries in Asia and associated with travel to these regions in non-endemic (Parry et al., 2002; Arjyal et al., 2011). MDR in these instances was characterized by resistance to the first-line drugs of treatment at that time which included chloramphenicol, ampicillin, and cotrimoxazole. Subsequently as was in the case of NTS, fluoroquinolones were introduced as the preferred drug of treatment for enteric fever (Arjyal et al., 2011). Resistance to this drug inevitably emerged with widespread use. Isolates with full resistance or reduced resistance to nalidixic acid and second generation fluoroquinolones such as ciprofloxacin and ofloxacin now dominate in Asia and emerging as the dominant isolates associated with enteric fever in many countries in Africa (Keddy et al., 2010; Kariuki et al., 2015a; Wong et al., 2015).

The epidemiology of fluoroquinolone resistant clones in sub-Saharan Africa is still an emerging story (Kariuki et al., 2004; Feasey et al., 2015; Wong et al., 2015). A travel-associated case of ciprofloxacin resistant *S*. Typhi was reported in South Africa with PFGE pattern 100% identical to pattern JPPX01.0026 in the Global PulseNet *Salmonella* Typhi (Keddy et al., 2010). However, ciprofloxacin-resistant salmonellae appear uncommon in Africa, at least within the available literature. Quinolone resistance and decreased-susceptibility to fluoroquinolones (ciprofloxacin MIC 0.12–1.0 μg/mL) are more prevalent (Table S2). Such strains are of public health importance because there is correlation between increasing MICs to fluoroquinolones and treatment failure (Parry et al., 2011). The reported prevalence rates of decreased susceptibility to ciprofloxacin from some African countries are 0.3% (Senegal) (Harrois et al., 2014) 1.6% (Ghana) (Nielsen et al., 2012), 4.3% (DRC) (Lunguya et al., 2013), and ~5% (Mozambique) (Mandomando et al., 2009) with reports also from Nigeria (Akinyemi et al., 2007) and Tanzania (Weill et al., 2006), for NTS; and for S. Typhi: 5.3% (South Africa) (Smith et al., 2010) and 60.4% (Kenya) (Kariuki et al., 2010). Some of these prevalences are high enough to warrant shifting to other antimicrobials but there are few alternatives in some of the least affluent African settings. There is an indication that different clones of these strains could circulate in Africa (Smith et al., 2010) and herald the emergence and dissemination of fluoroquinolone resistance. With the exception of reports from specific countries most notably Kenya (Kariuki et al., 2010) Malawi (Feasey et al., 2015), and South Africa (Keddy et al., 2010; Smith et al., 2010), a systematic analyses of lineages associated with drug resistance is yet to be reported. In the absence of a clearer picture, continued first-line usage and misuse of quinolones is more than likely to contribute the acquisition of drug resistance in typhoidal Salmonellae.

Fluoroquinolone resistant salmonellae are more common in animals compared to humans. A study in Senegal identified *Salmonella* in almost all bovine carcasses screened. That study reviewed earlier literature demonstrating that *Salmonella* in animals and meat is commonplace in many African settings (Stevens et al., 2006). Recently, the evolution and global (including Africa) dissemination of fluoroquinolone resistant *S*. Kentucky ST 198 X1-SGI 1 clone, which has poultry as the main reservoir has occured (Le et al., 2013). This epidemic clone is widespread in poultry in Nigeria and has persisted (Le et al., 2013; Raufu et al., 2014). Using ciprofloxacin resistance as a marker Le et al. (2013) reported that the strain is also probably present in pigs in Nigeria, although the two isolates identified in that study were not sequenced typed (Fashae and Hendriksen, 2014).

## Discussion

### Assessing clonality and tracking expansion

Methods that have been used to identify fluoroquinolone-resistant bacterial clones in Africa vary across the continent, making between-study and cross-country comparisons difficult (Quilici et al., 2010; Ismail et al., 2011; Marin et al., 2013). For toxigenic *V. cholerae* for example, serotyping and biotyping—both insufficient to confirm clonality—are only occasionally performed outside clinical reference laboratories (Quilici et al., 2010; Ismail et al., 2011). Genotyping the *ctxB* product (Quilici et al., 2010), MLSA (Marin et al., 2013), and PFGE (Ismail et al., 2011; Marin et al., 2013) have been used to infer clonality in African studies. PFGE has been employed in multiple studies but different protocols were used and the data are therefore non-comparable. Moreover, PFGE identifies different clusters within local clones and would not be a robust method for assessing clonality across Africa (Ismail et al., 2011).

One reason why so many methods have been employed is that so many methods abound. However the robustness, replicability, and inferences that can be made from available methods differ considerably. Methods based on phenotype or PCR amplification patterns have been more widely used in the decades following the evolution of fluoroquinolone resistance but have limited replicability across laboratories and in some cases, limited resolving power (Achtman, 1996). Serotyping is very popular but has the additional downside of being dependent on very labile reagents, a concern that is of particular importance in African laboratories. To ascertain the relatedness of pathogenic bacteria, methods that are specific enough to identify clusters of related isolates and of high resolution to distinguish isolates within these groups are ideal. Many classical molecular typing methods are based on the sequences of a few small loci of interest or on electrophoretic profiles dependent on particular nucleotide patterns. These principles respectively form the basis of multilocus sequence typing (MLST) and the comparison of PFGE patterns. Assessing MLST types and PFGE patterns are the methods most commonly used to infer clonality of sporadic or epidemic cases of enteric diseases in sub-Saharan Africa. They offer much greater reproducibility and portability than PCR-based or phenotypic methods and while they were initially pricey, set-up, and consumable costs have fallen dramatically in the last two decades. The major disadvantages of these methods are that they analyze a very small portion of the bacterial genome and different parts of a genome accumulate variation at different rates and/or are horizontally inherited. Finally, whilst still utilizable in specific circumstances, these methods lack the level of resolution needed to distinguish between very closely related bacteria.

The arrival of the so-called next-generation, high-throughput sequencing technologies makes it feasible to use WGS to identify and track clones of interest. The major intrinsic challenges so far have been high cost, inadequate infrastructure to maintain sensitive equipment and lack of technical capacity. Overcoming these surmountable challenges will present the opportunity to use the best-fit methods for studying the epidemiology and transmission of infectious diseases in such high burden areas in sub-Saharan Africa. It is increasingly important to situate these resources for WGS and analysis in areas with the most need to maximize gains. In the interim, a transitional advantage of WGS is that it can be used to generate comparative data for sequence based methods with less resolution, such as MLST.

The power and utility of WGS tools is exemplified in a recent study detailing the analyses of a global collection of *S*. Typhi (Wong et al., 2015). The *S*. Typhi H58 haplotype MDR clone has been shown to rapidly expand such as is the case in Malawi in which prior to 2011, sporadic Typhoid was caused by a diversity of clades. In their study, Wong et al. used a combination of WGS and Bayesian analyses to deduce the global population structure, possible intra- and inter-continental transmission pathways and an estimate of time of emergence of the most recent common ancestor of the current H58 global pandemic. These analyses confirmed the highly clonal nature of the H58 isolates, which were very similar to each other genetically (in fact, only ~6 SNPs separates any two H58 strains). They were also able to detect potential on-going epidemics in endemic countries. The H58 clones were estimated to have emerged in the late 1980's and are associated with high levels of drug resistance and reduced susceptibility to fluoroquinolones, due to mutations in DNA gyrase subunit and topoisomerase IV genes (Wirth, 2015; Wong et al., 2015). This is consistent with epidemiological and clinical data from the regions included in the study. This kind of analysis allows identification of genomic changes and acquisitions that underpin many observable phenotypes like increased infectivity, transmissibility or fitness when compared to closely related lineages of the same species. For example, Wong et al provide evidence to show that a chromosomally borne MDR locus and loss of an IncHI1 plasmid in a vast number of examined isolates may have increased the fitness of the H58 clones (Wirth, 2015; Wong et al., 2015).

### Exacerbating factors, intervention and research needs

Clonal expansion of fluoroquinolone resistance in enterics is broadly the consequence of antimicrobial pressure and bacterial and gene dissemination. Antimicrobial resistance is a “Tragedy of the Commons” evolutionary paradigm that is worsened by failure to appreciate the problem; that is the absence of a “recognition of necessity” (Hardin, 1968; Okeke, 2009). Thus, infrastructural, bacterial and human-related factors exacerbate the effects of antimicrobial pressure and bacterial exchange. Sub-par diagnostic infrastructure leads to incorrect estimation of true disease burden and veiling of significant resistant clones. The lack of national, regional and internationally coordinated antimicrobial stewardship leads to inconsistencies and non-uniformity in many reported cases; this makes it difficult to interpret data and to select truly representative studies.

#### Antimicrobial pressure

Antimicrobial pressure plays a large, important and potentially controllable role in the expansion of clonal resistance. Vien et al. showed that the prevalence of *qnr* resistance genes in gut Proteobacteria can increase following one course of antibiotic treatment (Vien le et al., 2012). In African countries, the contribution of pressure has not been measured, as antimicrobial use in humans and animals remains largely undocumented. Nonetheless, a high infectious disease burden warranting justifiable antimicrobial use, poor diagnostic test infrastructure and uptake and unregulated use of antibiotics combine to exacerbate the adverse effects that antimicrobial use has on resistance, and it's potential to select undesirable clones. While the community impact of antimicrobial use and overuse are often discussed, (Feasey et al., 2014) recently presented a case study illustrating that antimicrobial use can be associated with acquisition of fluoroquinolone resistance by a highly virulent clone of *Salmonella* Typhimurium in a treated patient. In that case study, recrudescence of a bloodstream infection isolate after acquisition of a resistance plasmid is the most likely explanation for death. The effects of antimicrobial pressure on the evolution of fluoroquinolone resistance extend beyond quinolone use. Evidence suggests that fluoroquinolone-resistant clones are selected and maintained by multiple antimicrobial classes, a feature that is easily explained by the fact that the successful clones in question are resistant to multiple antimicrobials. Interventions are required to prevent the over-prescription of antibiotics and preventing unsanctioned use in humans and livestock. Policies that address these issues have long been in existence but are inadequately implemented in most parts of Africa (WHO, 2001).

Antibiotic use strategies such as mixing and cycling have been a proposed tool for reducing antibiotic resistance. Such proposals are based on very few investigations, which give variable results as to their effectiveness (Brown and Nathwani, 2005; Abel zur et al., 2014). Studies with *S*. Typhi have shown that FQ resistance is not typically associated with fitness cost and suggests that FQ resistant strains would be naturally maintained even if fluoroquinolones use was reduced and so antibiotic cycling is unlikely to be beneficial (Baker et al., 2013). Even if mixing or cycling might be effective under some conditions, Africa currently has too few effective antimicrobial classes to effectively employ such techniques. In particular, withdrawing fluoroquinolones out of rotation limits treatment options for multiple life-threatening infections.

#### Dissemination

Enteric infections are principally attributable to poor sanitation, hygiene, food safety and access to potable water. Lack of such basic infrastructure contributes to endemicity in resource-poor settings and amplifies the prevalence of resistant strains in humans as well as animal and environmental reservoirs. Opportunities for bacteria to move from one colonized individual to another enhance the dissemination of resistant clones. In Africa, crowded cities and significant numbers of immunocompromised people are potential factors that could enhance the dissemination of clones. Interestingly, mathematical modeling to evaluate the impact of these risk factors found that increased pathogen transmission rates and longer durations of infectivity were even more important factors enabling spread, (Pitzer et al., 2015). Therefore, whilst unjustified antimicrobial use contributes to resistance, inadequate access to appropriate antibiotics when needed could also exacerbate the problem.

Travel has been associated with the dissemination of fluoroquinolone-resistant enteric clones in other parts of the world (Soraas et al., 2013). While there are reports pointing to export of resistant clones from African countries (Soraas et al., 2013), research does not appear to have determined whether and to what extent, resistant clones are imported into Africa. Research by Okoro et al. points to inter-country dissemination of multiply resistant non-typhoidal Salmonella in Africa but there are also very few studies documenting regional transmission as well (Okoro et al., 2012).

Clonal expansion of bacteria is often associated with selective advantage conferred by changes in bacterial genomes. These changes can be in the form the acquisition of chromosomal and/or plasmid-borne MDR determinants as well as mutations in genes targeted by antimicrobials, subsequently leading to the emergence and maintenance of fitter clones in animals and humans. This systematic review has shown that pandemic lineages within Africa are sometimes represented by different within-lineage clones than those elsewhere, pointing to bacterial factors that promote clonal expansion in select lineages. Host factors including presence of underlying conditions such immunosuppression (Gordon et al., 2007; MacLennan et al., 2008) and human migratory patterns attributable to social and economic pressures also contribute to the emergence and maintenance of MDR clones in Africa.

#### Surveillance

Our literature review identified very little research on fluoroquinolone-resistant enteric clones within Africa. There are however a large number of reports of fluoroquinolone resistant clones isolated in Europe and North America (Pitout et al., 2009; Soraas et al., 2013)that associate clone acquisition with travel to African countries (for example Pitout et al., 2009; Soraas et al., 2013). These “eyes of the hippopotamus” (Guerrant et al., 2005) reports point to under-detection and under reporting in Africa and the need for more local studies to estimate the true magnitude and extent of the problem. Our research also did not uncover significant information about resistance reservoirs, particularly in non-human hosts and the environment. What few animal data we did encounter however points to a large non-human reservoir of enteric organisms, many of them resistant, and the occurrence of these organism through the food chain. Studies reviewed for this paper were almost entirely small research studies on human isolates from a handful of laboratories. The location of these laboratories is the principal determinant of locations on the continent from which data are available. In order to understand resistance and track the emergence of clones, multi-center surveillance is required.

The European Antimicrobial Resistance Surveillance Network (EARS-Net) enables comparison between European countries (http://ecdc.europa.eu/en/activities/surveillance/EARS-Net/Pages/index.aspx). Recent analysis of the data has shown that an increase of fluoroquinolone consumption in Europe also increases the rate of resistance (Redgrave et al., 2014). To understand the scale of resistance in Africa, a similar model based on a regulated and coordinated surveillance system across Africa would be ideal. In contrast to the EU, where an obvious framework for coordination exists, it is less clear how to implement such a scheme across Africa. Potential frameworks include public as well as private sub-regional alliances and there are multicountry data from the Francophone Pasteur Institute Network (Breurec et al., 2013). Continental coverage cannot be obtained from either. Looking forward, a regional organization, perhaps the nascent African Society for Laboratory Medicine, or perhaps a purpose-initiated one, might be lead such an initiative. Alternatively, different laboratories and networks could feed data to a repository. As well as putting in proposed minimum reporting guidelines for research on antimicrobial resistance (Omulo et al., 2015), the surveillance system would also be expanded to include detection of clones and mechanisms of resistance that are causing resistance so that the spread can be tracked in real-time. A defined methodology for assessing clones to enable tracking would be needed so that different strains across Africa could be accurately compared and experience from Europe suggests that this is best achieved through sequence-based methods.

A network is only as good as the data it can collate from participating countries. Implementation of such a scheme in some countries would be difficult, given the state of existing infrastructure but existing institutions in many other countries that may be well-positioned to provide a local picture of resistance problems if the necessary coordination were to occur. As has been recently reported from Ghana, it is possible to set up a national surveillance system coordinated from the ministry of health (Opintan et al., 2015). The laudable Ghanaian initiative was not without its challenges, many of them linked to resource limitations, and the problem that most of the usable data came from only a handful of participating institutions, mostly referral hospitals. However inception is the first step in building a robust surveillance system and most African countries are yet to reach that point. In low-income African settings, it is predicted that susceptibility data will save lives and costs, compared to widespread used empirical therapy without local susceptibility information (Penno et al., 2015).

In addition to microbiological infrastructure and activity, support is needed for molecular studies that will identify the underlying genetic bases for resistance, including the existence and transmission of resistant clones. Comparison of isolates from within sub-Sahara Africa to those from outside the sub-region is important as recent studies have shown that clinical, cultural practices and socio-economic factors impact on the nature of circulating MDR clones in different parts of the world. Better antibiotic stewardship at local, country, and regional levels is needed in order to monitor and standardize protocols used for testing and clinical prescription. Much of the inquiry around antimicrobial resistance in African countries occurs within academic and non-governmental sectors, a likely contributor to fragmentation and limited coverage. Government participation and lead in public health initiatives such as strengthening of existing infrastructure and development of medical and research capacity remain a critical need.

## Author contributions

MC: Coordinated and collated systematic searches and references, evaluated eligible papers, performed systematic search, wrote sections of the manuscript. AA: performed systematic search, wrote sections of the manuscript, evaluated eligible papers, contributed to outlining the paper. CO, KF: performed systematic search, evaluated eligible papers, wrote sections of the manuscript. JO: evaluated eligible papers, edited sections of the manuscript and contributed to outlining the paper. IO: Initiated the project, performed systematic search, wrote sections of the manuscript, evaluated eligible papers, coordinated the writing.

### Conflict of interest statement

The authors declare that the research was conducted in the absence of any commercial or financial relationships that could be construed as a potential conflict of interest.
