# Supplementary material for: Fluoroquinolone-Resistant Enteric Bacteria in Sub-Saharan Africa: Clones, Implications and Research Needs
Source: Front Microbiol. 2016 Apr 22;7:558. doi: 10.3389/fmicb.2016.00558 (PMC4841292; doi:10.3389/fmicb.2016.00558)
Supplement: Supplementary file 1 [file Table1.DOCX]

Table S1 search terms

Searches were performed using all permutations of terms in list I AND terms in list 2 AND terms in list 3 on MEDLINE via PubMed and African Journals Online (AJOL)

| I | II | III |
| --- | --- | --- |
| Animal | fluoroquinolone resistance | Africa |
| *Campylobacter* | quinolone resistance | Angola OR Benin OR Botswana OR Burkina faso OR Burundi OR Cameroon OR Cape verde OR Central African Republic OR Chad OR Comoros OR Congo OR Cote d'Ivoire OR Democratic Republic of the Congo OR Equatorial Guinea OR Eritrea OR Ethiopia OR Gabon OR Gambia OR Ghana OR Guinea OR Guinea-Bissau OR Kenya OR Lesotho OR Liberia OR Madagascar OR Malawi OR Mali OR Mozambique OR Namibia OR Niger OR Nigeria OR Rwanda OR Soa Tome and Principe OR Senegal OR Seychelles OR Sierra Leone OR Somalia OR South Africa OR Swaziland OR Togo OR Uganda OR United Republic of Tanzania OR Zambia OR Zimbabwe |
| Commensal | ciprofloxacin resistance |  |
| Enterobactericeae | clone |  |
| *Escherichia coli* | clonal |  |
| *Salmonella* |  |  |
| *Shigella* |  |  |
| *Vibrio cholerae* |  |  |
|  |  |  |
