# Supplementary material for: Fluoroquinolone-Resistant Enteric Bacteria in Sub-Saharan Africa: Clones, Implications and Research Needs
Source: Front Microbiol. 2016 Apr 22;7:558. doi: 10.3389/fmicb.2016.00558 (PMC4841292; doi:10.3389/fmicb.2016.00558)
Supplement: Supplementary file 2 [file Table2.DOCX]

**Table S2:** Reports of reduced fluoroquinolone susceptibility and quinolone resistance in eligible studies

| **Study** | **Organism** | **Country** | **Reduced Susceptibility to FQ** | **Nalidixic acid Resistance** |
| --- | --- | --- | --- | --- |
| Kinana et al. 2007 | *Campylobacter coli* | Senegal | Reported: 15/36 | Reported: 14/36 |
| Kinana et al., 2006 | *Campylobacter jejuni* | Senegal | Reported: 19/46 | Reported: 22/46 |
| Cardinale et al. 2006 | *Campylobacter jejeuni/coli* | Senegal | Reported: 81/181 | Reported: 94/181 |
| Albrechtova et al., 2014a | *E. coli* | Angola | Reported: 3/19 | Reported: 2/19 |
| Namboodiri et al., 2011 | *E. coli* | Ghana | Reported: 23/193 | Reported: 40/193 |
| Feglo et al., 2013 | *E. coli* | Ghana | Reported: 28/29 | Reported: 97/156 |
| Kariuk et al. 2007 | *E. coli* | Kenya | Reported: 17/17 | Reported: 17/17 |
| Fortini et al., 2015 | *E. coli* | Nigeria | Reported: 26/32 | Not reported |
| Aibinu et al., 2012a | *E. coli* | Nigeria | Reported: 14/14 | Not reported |
| Lamikanra et al., 2011 | *E. coli* | Nigeria | Reported: 1/21 | Reported: 21/21 |
| Peirano et al., 2014 | *E. coli* | South Africa | Not reported | Not reported |
| Peirano et al., 2011 | *E. coli* | South Africa | Reported: 16/22 | Not reported |
| Gqunta and Govender, 2015 | *E. coli* | South Africa | Reported: 14/21 | Reported: 14/21 |
| Albrechtova et al., 2012 | *E. coli* | Kenya | Reported: 3/320 | Reported: 1/320 |
| Fortini et al 2011 | *E. coli* | Nigeria | Reported: 12/162 | Reported: 0/162 |
| Fortini et al 2015 | *E. coli* | Nigeria | Reported: 4/32 | Reported: 0/32 |
| Albrechtova et al., 2014b | *E. coli,* Enterobacteriaceae | Cote d'Ivoire | Reported: 44/192 | Reported: 9/192 |
| Rafai et al., 2015 | Enterobacteriaceae | Central African Republic | Reported: 55/65 | Not reported |
| Janatova et al., 2014 | Enterobacteriaceae | Central Africa Republic | Reported: 33/159 | Reported: 12/159 |
| Molla et al., 2007 | *Salmonella* | Ethiopia | Reported: 0/98 | Reported: 20/98 |
| Lunguya *et al.,* 2011 | *Salmonella* (NTS) | DRC | Reported: number of isolates not specified | Reported: 10/233 |
| Raufu et 2013 | *Salmonella* (NTS) | Nigeria | Reported: 3/149 | Reported: 0/149 |
| Fashae and Hendriksen, 2014 | *Salmonella* (NTS) | Nigeria | Reported: 5/229 | Reported: 0/229 |
| Raufu et al., 2009 | *S.* Hiduddify | Nigeria | Reported: 0/130 | Reported: 0/130 |
| Le et al., 2011 | S Kentucky | Nigeria | Reported: 0/197 | Reported: 0/197 |
| Raufu et al., 2014 | S Kentucky | Nigeria | Reported: 0/55 | Reported: 0/55 |
| Le et al., 2013 | S Kentucky | Nigeria | Reported: 4/70 | Reported: 0/70 |
| Weill *et al.,* 2006: | *S.* Kentucky | Kenya | Not reported | Reported: 4/17 |
| Holt *et al.,* 2008 | *S.* Typhi | Central Africa | Data not included in manuscript/study | Data not included in manuscript/study |
| Kariuki *et al.,* 2010 | *S.* Typhi | Kenya | Reported: 19/94 | Reported: 35/94 |
| Kariuki *et al.,* 2004 | *S.* Typhi | Kenya | Reported 48 isolates with increased MIC to both FQ and NA | Reported 48 isolates with increased MIC to both FQ and NA |
| Wong *et al.,* 2015 | *S.* Typhi | Malawi | Data not included in manuscript/study | Data not included in manuscript/study |
| Feasey *et al.,* 2015 | *S.* Typhi | Malawi | Reported: 1/112 | Not reported |
| Keddy *et al.,* 2010: | *S.* Typhi | South Africa | Not reported | Not reported |
| Smith *et al.,* 2010; | *S.* Typhi | South Africa | Reported: number of isolates not specified | Reported: 27/510 |
| Harrois *et al.,* 2014 | *Salmonella* (NTS) & *S*. Typhi | Senegal | Reported: number of isolates not specified | Not reported |
| Quilici et al., 2010) | *Vibrio cholerae* | Cameroon | Reported: 10/10 - MICs of ciprofloxacin  ranged from 0.25 to 0.5 mg/L. | Reported: 10/10 |
| Quilici et al., 2010) | *Vibrio cholerae* | Nigeria | Reported: 9/9 –  MICs of ciprofloxacin  ranged from 0.25 to 0.5 mg/L. | Reported: 9/9 |
| (Miwanda et al., 2015) | *Vibrio cholerae* | DRC | Reported: 0/1093 | Reported: >50%/1093 |
| (Mercy et al., 2014) | *Vibrio cholerae* | Kenya | Reported: 2/76 | Reported: 39/76 |
| (Marin et al., 2013) | *Vibrio cholerae* | Nigeria | Reported: 39/7620/20 | Reported: number of isolates not specified |
| (Mahmud et al., 2014) | *Vibrio cholerae* | Sierra Leone | Reported: 0/15 | Reported: 1/15 |
| (Ismail et al., 2011) | *Vibrio cholerae* | South Africa | Reported: 0/34 | Reported: 34/34 |
